# Influence of Detergent and Lipid Composition on Reconstituted Membrane Proteins for Structural Studies

**DOI:** 10.1021/acsomega.1c02542

**Published:** 2021-09-14

**Authors:** Mohammed Mouhib, Andrea Benediktsdottir, Caroline Svensson Nilsson, Celestine N. Chi

**Affiliations:** † Department of Medical Biochemistry and Microbiology, Uppsala University, BMC Box 582, SE-75123 Uppsala, Sweden; ‡ Institute of Chemical Sciences and Engineering, École Polytechnique Fédérale de Lausanne (EPFL), 1015 Lausanne, Switzerland; § Department of Medicinal Chemistry, Uppsala University, BMC Box 582, SE-75123 Uppsala, Sweden

## Abstract

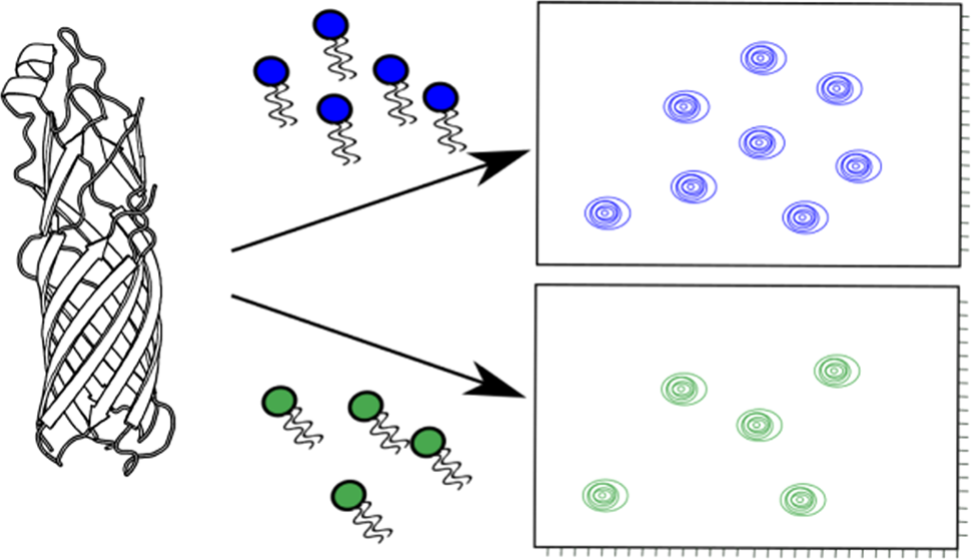

Membrane proteins are frequently reconstituted in different detergents as a prerequisite to create a phospholipid environment reminiscent of their native environment. Different detergent characteristics such as their chain length and bond types could affect the structure and function of proteins. Yet, they are seldom taken into account when choosing a detergent for structural studies. Here, we explore the effect of different detergents and lipids with varying degrees of double- or single-bond composition on ^1^H–^15^N transverse relaxation optimized spectroscopy spectra of the outer membrane protein W (OmpW). We observed changes in nuclear magnetic resonance chemical shifts for OmpW reconstituted in micelles, bicelles, and nanodiscs, depending on their detergent/lipid composition. These results suggest that a careful evaluation of detergents is necessary, so as not to jeopardize the structure and function of the protein.

## Introduction

**Figure 1:**
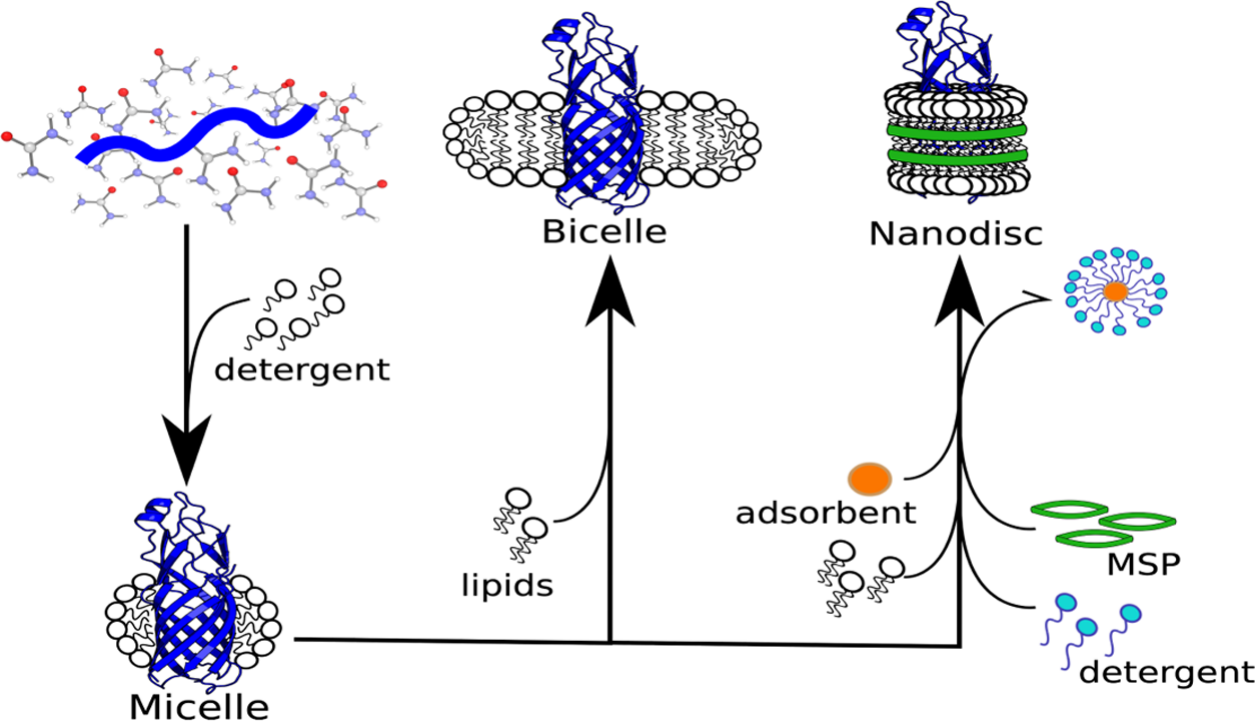
Stages of incorporating membrane proteins into different membrane mimetics. After a reasonable amount of the membrane of interest has been expressed (commonly in inclusion bodies), it is first denatured and refolded into micelles. Biophysical studies can be performed at this stage. Alternatively, the protein can be further incorporated into bicelles or nanodiscs as depicted above.

Integral membrane proteins are important for a multitude of cellular functions, such as the transport of metabolites and cell-to-cell signaling.^1^ Their relevancy in host–pathogen interactions makes their biophysical characterization relevant to the deep understanding of pathogenesis at a molecular level, as well as in drug design.^2^ Membrane proteins constitute 20–30% of all proteins and comprise a significant target for most drugs.^1^ However, very little structural information is available for this class of proteins. The process of obtaining decently folded and good-quality membrane proteins suitable for biophysical characterization and drug screening is often very frustrating, partly due to the poor stability and solubility of this class of proteins outside their lipid-rich environment. Moreover, the process is characterized by cycles of unfolding, refolding, and solubilization in different detergents, supposedly to generate an environment reminiscent of the native one^3^ (Figure 1). Often, different detergents are selected for the refolding process, the prerequisite being that the protein does not precipitate. Once this is achieved, the general fold of the protein is assessed by methods such as circular dichroism (CD).^6,7^ Because the detergents used for refolding are in direct proximity to the amino acid chains, there is no doubt that they can influence the structure, function, and dynamics of membrane proteins.^8−14^ Common detergents and phospholipids used for refolding, sometimes in combination with nanodiscs, include *n*-dodecylphosphocholine (DPC), 1,2-dihexanoyl-*sn*-glycero-3-phosphocholine (DHPC 6:0), 1,2-dimyristoyl-*sn*-glycero-3-phosphocholine (DMPC 14:0) and 1,2-dimyristoleoyl-*sn*-glycero-3-phosphocholine (DMoPC 14:1).^4^ However, extensive evaluation of the influence of detergents and lipids on refolded membrane proteins based on their nuclear magnetic resonance spectroscopy (NMR) spectra is rare.

Here, we investigated the effect of various detergents and phospholipids at the residue-specific level on the membrane protein OmpW by visualizing their effect on ^1^H–^15^N transverse relaxation optimized spectroscopy (TROSY) spectra. We observed changes in NMR chemical shifts among the micelle, bicelle, and nanodisc samples. Massive disappearance of signals and broadening of NMR lines were generally observed for the protein in DPC micelles. In contrast, DMPC 14:0 and DMoPC 14:1 nanodiscs, as well as DHPC 6:0/DMPC 14:0 bicelles yielded well-dispersed spectra. While the lipids used in nanodisc samples differ only in two single bonds within their chain, clear differences can be observed in their NMR spectra. These observations imply that both the detergents and lipids interact with the protein.

The epsilon ^1^Hε–^15^Nε proton shift of tryptophan side chains are often used as a marker to assess the overall fold of proteins containing tryptophan residues in their core. Of note is the fact that tryptophans in membrane proteins often reside in a lipid–water interface and are thought to play an anchoring role of membrane proteins to the membrane.^5^ This predisposition to the possibility of direct interaction with detergents or lipids makes tryptophan a useful proxy to evaluate the effect of these detergents/lipids on membrane proteins. OmpW contains five tryptophan residues, and epsilon resonances of all of these are observed in the DPC micelles. In contrast, only two or three of these could be observed in the well-dispersed spectra of bicelles and nanodiscs. These changes in the ^1^H-^15^N TROSY spectra and discrepancy in resolution or presence of resonance peaks between different parts of the spectra show that the impact of detergents and lipids on the membrane protein OmpW is complex at the residue-specific level.

Such an analysis highlights the importance of carefully evaluating and choosing detergents for structural and dynamic studies. Overall, the fact that we observed chemical shift changes for OmpW from different detergents/lipids implies that they have varying interactions with the protein and thus can influence the structure and dynamics of the protein and, consequently, its function. These results highlight that the choice of detergent or lipid for each protein has to be individually evaluated, as shown here for OmpW.

## Results and Discussion

### Two-Dimensional TROSY–Heteronuclear Single Quantum Coherence (HSQC) as a Measure for Structural Integrity of Refolded Proteins

**Figure 2:**
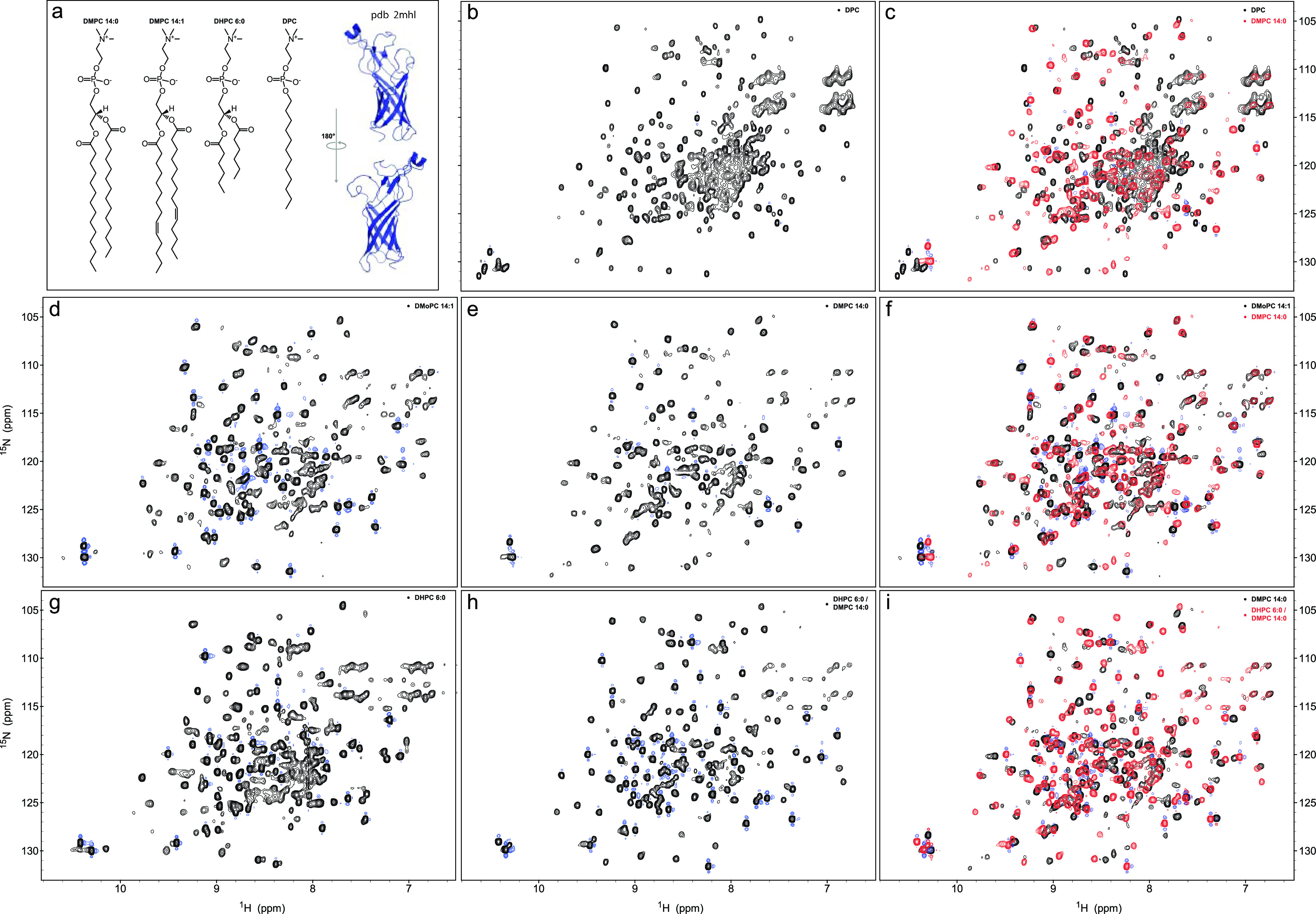
Overlay of 2D ^1^H–^15^N HSQC–TROSY spectra of OmpW refolded in different detergents. (a) Detergents and lipids used for the reconstitution into nanodiscs and bicelles, (b) OmpW refolded in DPC, (c) OmpW in DPC compared to OmpW in DMPC 14:0 nanodiscs revealed strong chemical shift changes due to a change in lipid environments, (d) OmpW in DMPC 14:1 nanodiscs, (e) OmpW in DMPC 14:0 nanodiscs, (f) comparison of OmpW in DMoPC 14:1 and DMPC 14:0 nanodiscs revealed chemical shift changes due to a different lipid environment, (g) OmpW in DHPC 6:0 detergent, (h) OmpW in DHPC 6:0/DMoPC 14:0 bicelles, and (i) comparison of OmpW in bicelles and DMPC 14:0 nanodiscs revealing strong chemical shift changes. All the spectra were acquired at 309 K.

To assess whether structural characteristics of OmpW remain similar across different detergents and membrane mimetics at a residue-specific level, we used NMR correlation spectroscopy. Therefore, we used OmpW refolded and reconstituted in DPC, DHPC 6:0, DMPC 14:0 nanodiscs, DMoPC 14:1 nanodiscs, and DHPC 6:0/DMPC 14:0 bicelles in 2D ^1^H–^15^N TROSY–HSQC NMR-type experiments. OmpW gave overall well-dispersed TROSY–HSQC spectra in all sampled detergent, detergent–lipid, and nanodisc combinations. However, on detailed inspection, clear chemical shift differences among the various detergents and lipids could be observed (Figure 2). OmpW reconstituted in DPC micelles gave signals for all of the tryptophan epsilon ^1^Hε–^15^Nε bond pairs, indicating that the protein is well folded, despite the presence of some broadening in the middle of the spectrum (Figure 2a). The outcome was different when OmpW was reconstituted in DHPC 6:0, DMPC 14:0, or DMoPC 14:1 (Figure 2d–g). Although the spectra were more dispersed, only two to three of the five epsilon tryptophan resonances were visible. A lot of resonances were also missing in DMPC 14:0 nanodiscs, indicating a significant impact of the lipid on the backbone amide resonances. There was a slight rescue of the signals when OmpW was reconstituted in bicelles using the same lipid (Figure 2h). In this case, the spectrum became more dispersed and more resonances reappeared. However, only three of the five epsilon resonances from the tryptophan side chains were visible.

**Figure 3:**
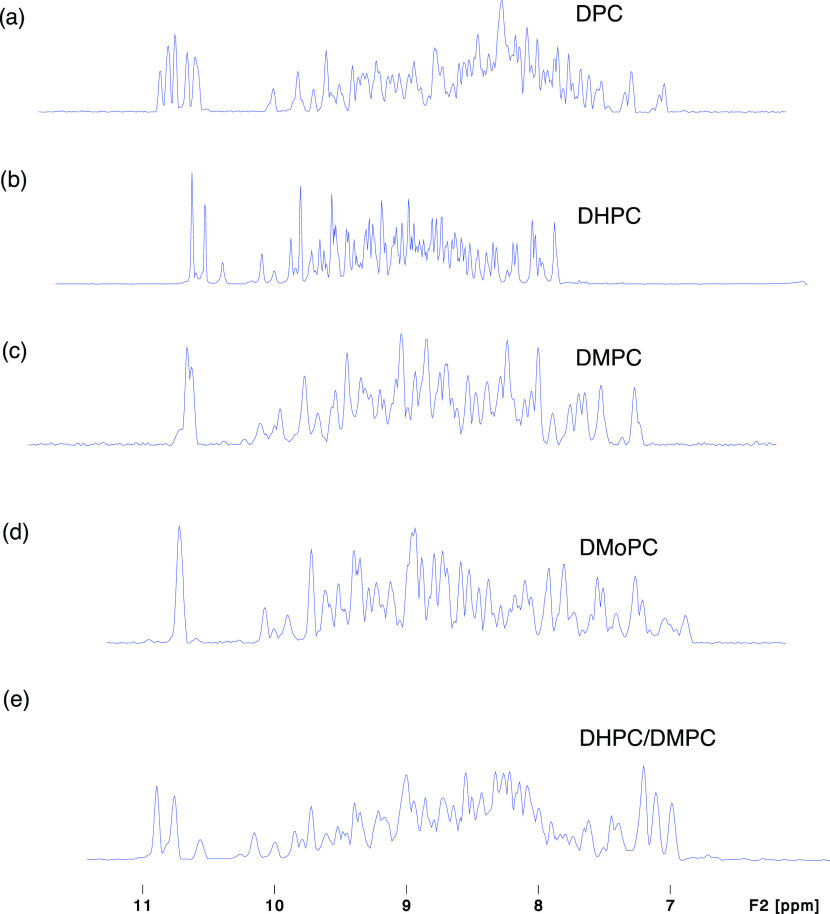
Display of 1D spectra of OmpW solubilized in (a) DPC, (b) DHPC, (c) DMPC-nanodics, (d) DMoPC-nanodics, and (e) DHPC/DMPC-nanodics.

While these differences in the side chains of W resonances might indicate a change of the OmpW structure and dynamics, the presence of detergents and lipids per se might also influence other parameters. For instance, the overall tumbling times of OmpW will vary between micelles, bicelles, and nanodiscs. In addition, varying protein concentrations could also cause significant differences in recorded spectra. To evaluate whether the observed change in tryptophan side-chain resonances was caused by these effects, we compared the average intensity profiles of the visible tryptophan peaks to the rest of the protein chain (Figure 3). We observed that relative changes vary between intensity ratios of 1.5 to up to 5. Given that the protein concentration was constant (0.2 mM) in all experiments, the relative loss in signal of some tryptophan side chains is due to broadening of these signals induced by the presence of detergents/lipids. This may partly be caused by direct interaction of tryptophan residues in particular with detergents/lipids.^5^ Clear differences could be seen between the TROSY–HSQC profiles of DMPC 14:0 and DMoPC 14:1 nanodiscs, indicating that not only the chain length but also the degree of saturation of phospholipids and the entailed change in their physicochemical properties (for example, their melting temperature) can influence the structural properties of membrane proteins (in this case OmpW). These results are intriguing, and judging from the TROSY–HSQC spectra, all detergent and lipid types seem to interact differently with OmpW. Detergents have been noted to affect the function of membrane proteins^7−9^ as well as influence their dynamics.^10^ It has also been suggested that the solubility of a membrane protein in a particular detergent or detergent–lipid does not translate to native structure and stability. In addition, it has been proposed that a detergent or detergent–lipid combination suitable for a membrane protein cannot be generalized to other membrane proteins.^6,7^ This means screening of a handful of detergent and detergent–lipid classes is needed to get the right combination for a specific membrane protein of interest.

However, in practice, only 1–2 detergents or lipid types are evaluated. There have been a few cases where the influence of combinations of detergent and detergent-lipids (micelles, bicelles, and nanodiscs) has been compared.^10,11^ These studies found a profound influence of detergents and detergent–lipid combinations on the dynamics of a membrane protein (OmpX), as were evident from their NMR 2D-TROSY–HSQC spectra. However, it was not clear if changes in detergents/lipids such as bond characteristics will also influence the membrane protein structure. Here, we report a comparative analysis of OmpW in different detergents and detergent–lipid containing similar chain lengths but differing in their degree of bond saturation or chain substitution. We found that the NMR spectra, which most often reflect the structure of the protein, were different for OmpW solubilized in detergents/lipids similar in chain length but differing in these characteristics. This implies that when choosing detergents/lipids for structural studies, their degree of bond saturation or chain substitution should be taken into consideration in addition to chain lengths and other substitutions. The case of OmpW as seen in this study may be unique and highlights the importance of detergents/lipids on the refolding of membrane proteins and the use of NMR chemical shift as a proxy of membrane protein integrity to be verified on a case-by-case basis.

## Materials and Methods

### Expression and Purification of OmpW

OmpW was expressed in *E. coli* Bl21* in a minimal M9 medium made up of 99.6% D_2_O and containing 2 g d-glucose-*d*_7_. Initially, a single colony was grown in a preculture made up of 10 mL of LB medium dissolved in 99.6% D_2_O until an OD_600_ of 0.8 was reached. Further, 5 mL of this preculture was added into 400 mL of 99.6% D_2_O M9 medium. This was then grown overnight (14 h). The next day the 400 mL culture was added into 1600 mL of 99.6% D_2_O M9 medium and the culture was incubated on a rotating shaker at 37 °C. Recombinant protein expression was induced by the addition of 1 mM IPTG at an OD_600_ of 0.8. Cells were then allowed to grow for additional 5 h and were harvested by centrifugation. Cells were incubated with 1 tablet of protease inhibitor (EDTA-free) for 30 min at 4 °C and lysed by sonication (35 psi, 6 min), followed by centrifugation at 47 808*g* and 4 °C for 1.5 h. The pellets were resuspended in buffer containing 20 mM Tris-HCl pH 8, 0.5 M NaCl, 0.5 v/v Triton X-100 with further shaking at 37 °C for 1h. Subsequently, the suspensions were centrifuged (4 °C, 8000*g*, 35 min), pellets were resuspended (20 mM Tris-HCl pH 8, 0.5 M NaCl), and centrifuged again (4 °C, 8000*g*, 35 min), and then finally resuspended in 50 mL of 6 M GdmCl, 20 mM Tris-HCl pH 8. OmpW-containing fractions were pooled and stored at −20 °C for later use or refolded as below. MSPΔH5 (nanodiscs) were expressed in *E. coli* using LB media and purified as previously described.^6^

### Refolding of OmpW in DHPC 6:0 or DPC Detergent and Reconstitution in a Nanodisc and Bicelle

OmpW was refolded by a dropwise (0.3 mL/min) addition of 5 mL of protein solution (6 M GdmCl) into 50 mL of refolding buffer (0.5 mM DPC or 30 mM DHPC 6:0, 50 mM Tris, pH 8, 100 mM NaCl, 500 mM l-arginine) and was afterward dialyzed three times against 4 L of 20 mM Tris, pH 7.4, 100 mM NaCl, for a total time of 14 h. OmpW was concentrated using a 10 kDa cutoff Amicon centrifugal filter.

OmpW was reconstituted in a DMPC 14:0 or DMoPC 14:1 nanodisc using the MSPΔH5 construct and at an assembly ratio of OmpW:MSPΔH5:lipids of 1:2:80 and an OmpW (DPC) concentration of 200 μM. The mixture was gently shaken overnight at 27 or 20 °C, with subsequent addition of 1 g Biobeads SM-2 to the assembly volume for 4 h. Afterward, the solution was separated from the Biobeads by low-speed centrifugation. Nanodisc complexes were purified by size-exclusion chromatography using a Superdex 200 10/300 GL column, from which the main fractions were collected and concentrated using a 30 kDa cutoff Amicon centrifugal filter.

OmpW was reconstituted in a DHPC 6:0/DMPC 14:0 bicelle using a *q* ratio of 0.5 (DMPC 14:0/ DHPC 6:0), whereby the DHPC 6:0 concentration was estimated by 1d-NMR spectroscopy comparing the intensity of CH_2_ groups to a standard DHPC 6:0 concentration. After estimation of the DMPC 6:0 concentration, 0.5 times DMPC 14:0 was added and going several times through the transition temperature of the DMPC 14:0 (25 °C) by cooling (4 °C) and warming cycles (35 °C) resulted in the formation of the bicelle.

### NMR Spectroscopy

All NMR experiments were collected on a 600 MHz Bruker Advance Neo equipped with a TCI cryoprobe (TR-1H &19F/13C/15N 5 mm-EZ) at the Uppsala NMR center and on a 600 MHz Bruker Advance III spectrometer equipped with a TCI cryoprobe. The buffer for the NMR experiments was 20 mM Tris, pH 7.4, and 100 mM NaCl, and the experiments were done at 310 K. TROSY–HSQC-based ^1^H–^15^N with nonuniform (b_trosyetf3gpsi.2) and uniform (trosyetf3gpsi) sampling was used for all 2D experiments. For nonuniform sampling, a sampling rate of 50% was used. A total of 4–512 scans for the micelle or 256–512 scans for the vesicle samples were acquired. A total of 256 points in the F1 and 768 points in the F2 were collected. The spectra width in F1 was 38 ppm and the middle of the spectrum was set at 118 ppm. In the F2, the middle was set at the water resonance (4.7 ppm) with a spectral width of 18 ppm. The relaxation delay was 0.7 s. All spectra were processed with Topspin 4.0 software and analyzed using the program CcpNmr.^12^
